# Niacin – a scoping review for Nordic Nutrition Recommendations 2023

**DOI:** 10.29219/fnr.v67.10299

**Published:** 2023-12-12

**Authors:** Riitta Freese, Vegard Lysne

**Affiliations:** 1 Department of Food and Nutrition, University of Helsinki, Helsinki, Finland; 2 Norwegian Institute of Public Health, Oslo, Norway

**Keywords:** niacin, vitamin B3, nicotinic acid, nicotinamide, nicotinamide adenine dinucleotide, tryptophan, requirement, nutrition recommendation, Nordic countries

## Abstract

Niacin is the precursor to pyridine nucleotides NAD (nicotinamide adenine dinucleotide) and NADP (nicotinamide adenine dinucleotide phosphate). Niacin (vitamin B_3_) is the common term for nicotinic acid, nicotinamide and derivatives that exhibit the biological activity of nicotinamide. Furthermore, the indispensable amino acid tryptophan is the substrate for *de novo* synthesis of NAD. Thus, the requirements and intake of niacin are expressed as niacin equivalents (NE).

The focus of interest for niacin over the last decade has primarily been on pharmacological doses of nicotinic acid as a lipid-lowering agent and other NAD precursors as potential enhancers of cellular NAD^+^ concentrations. None of these studies, however, makes a useful contribution to understanding dietary requirements in healthy populations.

The requirement for niacin is estimated based on the relationship between intake and biochemical indices of niacin status, primarily urinary excretion of nicotinamide metabolites.

## Popular scientific summary

- Niacin, also known as vitamin B_3_, includes nicotinic acid and nicotinamide, which have essential roles in numerous biochemical reactions in the body.
- Meat (beef, poultry), fish, dairy, legumes (including peanuts), some cereals, and baker´s yeast are high in niacin
- The main indicator of niacin status in adults is the urinary excretion of niacin metabolites
- The classical niacin deficiency disease, pellagra, is characterized by diarrhea, dermatitis, dementia, and death.
- Niacin is currently of pharmacological interest for potential prevention or treatment of metabolic diseases

Niacin (vitamin B_3_) is the common term for nicotinic acid (pyridine-3-carboxylic acid), nicotinamide (pyridine-3-carboxamide), and derivatives that exhibit the biological activity of nicotinamide (1, 2).

Niacin is the precursor to pyridine nucleotides NAD (nicotinamide adenine dinucleotide) and NADP (nicotinamide adenine dinucleotide phosphate). In the form of NAD(H) and NADP(H), niacin is involved in more reactions in the body than any other vitamin derivatives. In addition to direct vitamin precursors for NAD production (niacin), the indispensable amino acid tryptophan is the substrate for *de novo* synthesis of NAD. The requirements and intake of niacin are expressed as niacin equivalents (NE). On average, 60 mg of dietary tryptophan is estimated to yield 1 mg niacin (60 mg tryptophan = 1 mg NE). The term vitamin B_3_ can be defined as the dietary NAD precursors other than tryptophan (3).

Niacin and the derivatives NAD(P) as well as the amino acid tryptophan are widely distributed in foods of animal and plant origin. Bioavailability from some plant sources is low. The amount of bioavailable niacin needed to prevent the deficiency disease pellagra is small, and the average requirements (ARs) for adults are in the scale 12 to 15 mg/d (1). Disturbances of NAD homeostasis at the cellular level have been shown to be associated with aging and many metabolic diseases. Therefore, much interest is currently directed to the possibilities to prevent or treat different metabolic diseases with pharmacological doses (in the order of 100s or 1000s mg/d) of different forms of niacin (4).

The aim of this scoping review is to describe the totality of evidence for the role of niacin for health-related outcomes as a basis for setting and updating dietary reference values (DRVs) (Box 1).

Box 1 Background papers for Nordic Nutrition Recommendations 2023

- This paper is one of many scoping reviews commissioned as part of the Nordic Nutrition Recommendations 2023 (NNR2023) project (5).
- The papers are included in the extended NNR2023 report but, for transparency, these scoping reviews are also published in Food & Nutrition Research
- The scoping reviews have been peer reviewed by independent experts in the research field according to the standard procedures of the journal
- The scoping reviews have also been subjected to public consultations (see report to be published by the NNR2023 project)
- The NNR2023 committee has served as the editorial board
- While these papers are a main fundament, the NNR2023 committee has the sole responsibility for setting dietary reference values in the NNR2023 project

## Methods

This review follows the protocol developed within the NNR2023 project (5). The sources of evidence used in the scoping review follow the eligibility criteria described in the paper ‘The Nordic Nutrition Recommendations 2022 – Principles and methodologies’ (6). No *de novo* NNR2023 systematic reviews relevant for this scoping review were conducted (7), but one existing qualified systematic review (8) was identified by the NNR2023 Committee.

The main literature search for this scoping review was performed on July 2, 2021 in MEDLINE with a search string: (Niacin[MeSH Terms] OR Niacinamide[MeSH Terms] OR Niacin[Title] OR “nicotinamide riboside”[Title] OR nicotinamide[Title]) AND review[Publication Type] AND (“2011”[Date - Publication] : “3000”[Date Publication]) AND humans[Filter]. The number of hits was 856. Based on the title, 105 abstracts were checked, and 10 articles were considered relevant (9–18). Of these 10 articles, five were systematic reviews (9, 10, 12, 16, 17).

To update the Physiology and Health outcomes sections, relevant up-to date textbooks (3, 19–21) were consulted. Furthermore, a literature search in MEDLINE with a search string (NAD[Title]) AND review[Publication Type] AND (“2011”[Date - Publication] : “3000”[Date - Publication]) AND humans[Filter] was carried out on July 5th 2021. The search returned 166 references of which five were considered relevant (4, 22–25).

The previously published niacin recommendations or opinions from the Institute of Medicine (from 2015 onwards National Academy of Sciences, Engineering, and Medicine, NASEM) (2, 26) and the European Food Safety Authority (EFSA) (27–29) have been used in the present review, as well.

A separate literature search was carried out on vegan/vegetarian diets and niacin status: (Niacin[MeSH Terms] OR vitamin B3) AND (vegan[title) OR vegetarian[title]) AND (“2011”[Date - Publication] : “3000”[Date - Publication]) AND humans[Filter] (July 5th 2021). One systematic review (30) was considered relevant. After the public consultation (May–June 2022), another systematic review was included in the present scoping review (31).

No strong evidence was identified in scientific literature since 2012 that likely would cause a change in DRV. Neither was any topic related to a substantial health concern in the Nordic or Baltic countries identified. An updated search was performed on August 1st, 2022. The scoping review authors and the NNR committee are aware of these publications, however, the information in these publications does not change the judgment regarding the setting of DRVs.

## Physiology

Rich dietary sources of preformed niacin are meat (beef, poultry), fish, dairy, legumes (including peanuts), some cereals, and baker´s yeast (19, 21). In meat, niacin is mainly bound to NAD and NADP (3). Milk is a source of nicotinamide riboside (32). The coenzyme forms (NAD, NADP) are hydrolysed by intestinal phosphatases and NAD glycohydrolases to release nicotinamide and nicotinamide riboside, and the latter is further hydrolysed to nicotinamide (19). In plant products, especially in cereal grains, niacin is found mainly as nicotinic acid that is often bound to proteins, glycopeptides, or polysaccharides and is poorly available (20). Food processing (alkaline treatment) may increase the bioavailability of the nicotinic acid in cereals.

**Fig. 1:**
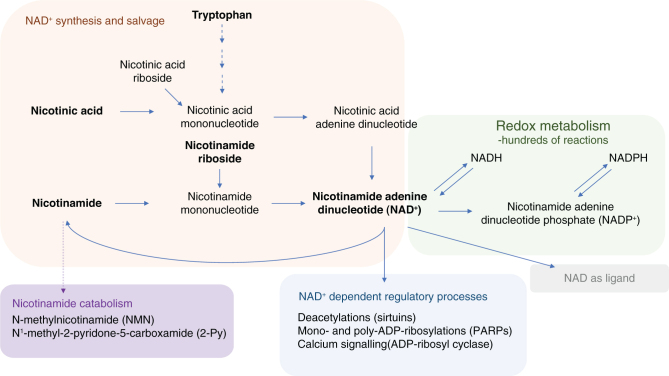
NAD synthesis, functions, and metabolism. The dietary NAD precursors (mainly nicotinic acid, nicotinamide, nicotinamide riboside, or tryptophan) contribute to NAD synthesis via different pathways (*de novo*, Preiss-Handler, and salvage pathways). NAD(H) and NADP(H) are used in the cells in hundreds of oxidation-reduction reactions in the energy metabolism and various synthesis/degradation systems. NAD^+^ is also used as a substrate in enzyme systems that control, for instance, DNA repair, transcriptional regulation, circadian rhythms, mitochondrial homeostasis, and calcium signaling. Furthermore, NAD^+^ serves as a ligand of purinergic P2Y receptors that are involved in the regulation of the activities of visceral smooth muscles and immune cells. The cellular pool of NAD^+^ is tightly balanced by the regulation of NAD^+^ synthesis and its breakdown by NAD^+^-consuming enzymes. The subcellular localization of the reactions or the enzymes needed are not presented in the figure (Based on 3, 4, 11, 25).

Nicotinic acid and nicotinamide are absorbed by carrier-mediated mechanisms and passive diffusion. The intestinal niacin transporters have not yet been fully clarified. Some diffusion takes place in the stomach, but niacin absorption is more effective in the small intestine (20). The main form of niacin in serum is nicotinamide. Red blood cells contain a circulating pool of pyridine nucleotides. Different NAD precursors are taken in the cells by diffusion or transporter-mediated processes (20, 25). Liver is the center of niacin metabolism, and, for instance, large part of NAD synthesis from tryptophan and the conversion to excreted metabolites takes place in the liver. The excretion route of niacin metabolites is urine (20). The main pathways of niacin and NAD metabolism are shown in Fig. 1.

In general, it is assumed that 60 mg of tryptophan is needed for *de novo* synthesis of 1 mg of NAD. The conversion efficiency depends on tryptophan status rather than on niacin status. The amino acid is preferably used for protein synthesis under conditions of limiting amounts of tryptophan, and the conversion efficiency is reduced below the commonly used NE ratio (19, 26, 27). Thus, low tryptophan intake, deficient tryptophan transport (e.g. Hartnup´s disease) or conditions associated with increased tryptophan metabolism decrease the conversion and increase the need for preformed niacin. Furthermore, adequate intakes of the micronutrients needed as cofactors in the *de novo* pathway (iron, riboflavin, vitamin B6) are needed. The body has a limited capacity for storing niacin nucleotides and deficiency symptoms can occur after 50–60 days of consuming a low-niacin, corn-based diet (26).

Cellular NAD homeostasis is the balance between synthesis, consumption, and regeneration of NAD. The homeostasis may be disturbed due to mutations in enzymes involved in NAD synthesis. Furthermore, NAD metabolism is disturbed in many diseases and in dietary deficiency of NAD precursors (25). Cellular NAD^+^ levels decline with aging. The strongest evidence by far is from animal models, but evidence is accumulating that the decline takes place also in humans (4, 23). However, the mechanisms driving NAD^+^ depletion during aging are not yet fully understood.

A systematic review indicated that niacin intake was lower in vegans than in other diet groups in several studies, and niacin was pointed out as one of the vitamins of concern in vegan diets (30). Another systematic review (31) assessed the results from 15 studies. In those analyses, vegetarians had lower intakes of niacin compared with vegans or so-called meat-eaters (consumption of meat at least once per week). However, in all dietary pattern groups, the mean intake of niacin was above the estimated average requirements (EARs) 11 or 12 mg/d for women and men, respectively (2).

## Assessment of nutrient status

Niacin status can be measured by urinary excretion of nicotinamide metabolites, including N’-methylnicotinamide (NMN) and methyl pyridone carboxamides such as N-methyl-2-pyridone-carboxamide (2-Pyr) (Fig. 1). The excretion of these metabolites decreases in niacin deficiency (27). Urinary excretion of metabolites has been reported to increase sharply by intake higher than 11 mg NE/day, which has been suggested to reflect saturation of body stores (27).

Biochemical niacin status can be measured from tissue (cellular) NAD and NADP concentrations. Cellular NADP concentrations are stable during deficiency, but NAD concentrations decline. The ratio of NAD to NADP in whole blood (red blood cells) can be used as a marker of functional niacin status (3). More versatile reflection of niacin status will probably be obtained with metabolomics in the future (3).

## Dietary intake in Nordic and Baltic countries

Niacin occurs in foods such as meat, fish, dairy, legumes (including peanuts), cereals, and baker´s yeast (19, 21). Plant foods primarily contain nicotinic acid, while animal foods primarily contain nicotinamide. Protein-rich foods also contribute to the niacin intake through endogenous conversion from tryptophan, and 60 mg tryptophan is equivalent to 1 mg NE.

The diet among adults (>18 years) in the Nordic countries provides 29–31 and 39–41 mg NE for women and men, respectively. This corresponds to relative intakes of 3.5–4.3 and 3.6–4.4 mg NE/MJ and 14.6–17.7 and 15–18.1 mg NE/1000 kcal, for women and men, respectively.

Tryptophan makes up approximately 1% of the amino acids in dietary proteins (3). The average protein intake in the Nordic countries of 92–112 and 72–81 g/day for men and women, respectively, and this would provide 12–19 mg NE/day (33).

## Health outcomes

### Deficiency

The classical niacin deficiency disease pellagra is characterized with four D’s: diarrhea, photosensitive dermatitis, dementia, and, if not treated, death. The various symptoms reflect the multiple roles of niacin/NAD in the whole-body metabolism (20).

Pellagra is mainly observed in populations consuming a diet predominantly based on maize or other cereals with a low protein content and low bioavailability of niacin. Few controlled studies, with few subjects, have investigated the effects of niacin-restricted diets (26, 34). In one controlled study, pellagra developed at an intake of 8.8 mg NE/d (26). In two other studies, no clinical symptoms were seen in subjects with an intake of 9.2–12.3 mg NE per day, which is the equivalent to about 1 mg NE/MJ (26). Niacin deficiency and clinical signs of pellagra are rare in high-income countries but may appear in association with anorexia nervosa, alcoholism, acquired immunodeficiency syndrome, cancer, and chemotherapy (18, 21).

### Upper intake levels and toxicity

There are no studies indicating adverse effects of consumption of naturally occurring niacin in foods. However, adverse effects may result from excess intakes from dietary supplements, fortified foods, and pharmacological agents. Intakes of nicotinic acid, but not nicotinamide, as a supplement or fortificant in the range of 30 mg/d to 1000 mg/d can result in mild symptoms such as flushing. Higher intakes have been reported to induce liver damage. The EU Scientific Committee for Food (28) has set an upper limit for nicotinic acid of 10 mg/d and for nicotinamide of 900 mg/d for adults. These levels were also used in the NNR 2012. A more recent meta-analysis evaluating dose-dependent adverse effects of nicotinic acid and nicotinamide supplementation suggested reconsideration of the UL for nicotinic acid supplements and differentiation between healthy and unhealthy individuals (12). In this meta-analysis, a benchmark-dose method, considered by EFSA as a more advanced method compared to using NOAEL/LOAEL (35), was applied to estimate the intake level associated with a 5% incidence of adverse effects. Maximum intake levels considerably higher than the current UL were reported, and different estimates were found depending on whether the population was healthy or diseased. Considering the other NAD precursors, the EFSA Panel on Nutrition, Novel Foods and Allergens has concluded that nicotinamide riboside chloride as a novel food used in food supplements for the general healthy adult population at levels up to 300 mg/d, and an intake up to 230 mg/day is safe for pregnant and lactating women (29).

Several meta-analyses of clinical trials on niacin supplementation have reported results on adverse effects. Compared to placebo, adults with or at risk of cardiovascular disease receiving niacin therapy (500–4000 mg/day) were more likely to stop the treatment due to side effects of which the strongest risk was reported for flushing, pruritus, rash, gastrointestinal symptoms, and new-onset diabetes (17). Increased risk of flushing was also reported in patients with renal disease treated with pharmacological doses of niacin (375–1500 mg/day) (10).

### Cardiovascular disease

Therapeutic doses of nicotinic acid have been shown to increase serum high-density lipoprotein (HDL) cholesterol, and lower serum low-density lipoprotein (LDL) and total cholesterol concentrations (3, 9). Further, in a systematic review and meta-analysis of seven randomized controlled trials (RCTs), including 441 subjects, Sahebkar reported that niacin supplementation improved endothelial function, expressed as a weighted mean (95% confidence interval [CI]) increase in flow-mediated dilation of 1.98 (0.91, 3.05) % (16). However, the effect on intermediate risk factors does not seem to transfer to hard clinical endpoints. In a systematic review and meta-analysis of 23 RCTs published between 1968 and 2015, including 39,195 participants, Schandelmeier et al. (17) reported that supplementation with nicotinic acid (median dose 2 g/day) did not substantially influence total mortality (RR [95% CI] 1.05 [0.97, 1.12]), cardiovascular mortality (1.02 [0.93, 1.12]), non-cardiovascular mortality (1.12 [0.98, 1.28]), acute myocardial infarction (0.93 [0.87, 1.00]), or stroke (0.95 [0.74, 1.22]) (17).

### Cancers

NAD^+^ is involved in the regulation of genomic stability, and NAD precursors are also of interest in terms of cancer prevention. At present, most evidence has been accumulated on the possible preventive effects of pharmacological doses of nicotinamide on non-melanoma skin cancers (13). Three cohort studies of female populations did not find an association between niacin intake, assessed by a food frequency questionnaire, and five major cancers (breast, endometrial, ovarian, colorectal, and lung) (8).

### Other health outcomes

Rodent and human studies have shown that alterations in NAD^+^ homeostasis may be involved in most age-related diseases, including neurodegeneration, diabetes, and cancer (24). Thus, therapeutic approaches to boost cellular NAD^+^ concentration, including NAD precursor supplementation with doses higher than achievable from normal diet, are under active investigation. A large part of the evidence thus far comes from animal experiments, and evidence from human efficacy trials has only started to accumulate (4, 14, 15, 22, 25). The association between dietary niacin intake and cognitive decline has been reported in two prospective cohort studies and a cross-sectional study, collectively suggesting that higher niacin intakes could have protective effects on the development of Alzheimer’s disease and cognitive decline (8).

Some clinical trials have indicated that niacin or nicotinamide supplementation may reduce serum phosphate in patients with chronic kidney disease receiving dialysis therapy. In a systematic review and meta-analysis of five RCTs, in total including 230 patients, He et al. (10) reported that niacin or nicotinamide in doses ranging from 375–1000 mg/day reduced both serum phosphorus (weighted mean difference [95% CI] −0.88 [−1.19, −0.57]) and the calcium x phosphorus product (−9.15 [−13.23, −5.08]) (10).

## Requirement and recommended intakes

The main criteria used to determine niacin requirements in adults are the urinary excretion of the nicotinamide metabolites, which sharply decreases when niacin intake is inadequate. Based on studies of niacin-deficient diets, the excretion of at least 1 mg of N1-methylnicotinamide/day has been considered to reflect a sufficient intake (2, 27). In situations of low tryptophan intake, deficient tryptophan transport (e.g. Hartnup´s disease) or conditions associated with increased tryptophan metabolism (e.g. carcinoid syndrome), conversion from tryptophan is decreased and the need for preformed niacin may be higher. Inadequate iron, riboflavin, or vitamin B6 status decreases the efficiency of the *de novo* pathway (26, 27).

In NNR 2012 (1), the AR was estimated at 1.3 mg NE/MJ, based on older depletion-repletion studies using urinary excretion of niacin metabolites as the main criteria (34). The recommended intake (RI) was set at 1.6 mg NE/MJ, corresponding to RIs of 16–19 and 13–15 mg NE/day for adult men and women, respectively. It was emphasized that for people on low-energy diets (below 8 MJ/day), the RI of 1.6 mg NE/MJ may not be sufficient, and it was therefore recommended that niacin intake should not be less than 13 mg NE/day. For pregnant and lactating women, an extra 1–2 and 4–5 mg NE/day, respectively, was recommended. The RIs for children over 6 months of age were extrapolated from the adult values.

The U.S. DRVs were based on urinary excretion of niacin metabolites and expressed in absolute intakes (2). The EAR was set to 12 and 11 mg NE/day, and the recommended daily allowance (RDA) was 16 and 14 mg NE/day for adult men and women, respectively. It was emphasized that individuals with Hartnup’s disease, liver cirrhosis, carcinoid syndrome, malabsorption, and patients on long-term treatment for tuberculosis or undergoing dialysis treatment were likely to require more niacin. Also, pregnant women carrying more than one fetus or breastfeeding multiple children may need extra niacin.

EFSA (27) considers urinary excretion of niacin metabolites as the main criteria for setting DRVs. The AR for adults was kept at 1.3 mg NE/MJ, and by applying a coefficient of variation of 10%, the population RI was set to 1.6 mg NE/MJ for both sexes and all ages. They concluded that there was no evidence that the niacin requirement in pregnancy and lactation increased above what was determined by the extra energy requirement, and the same RI of 1.6 mg NE/MJ should apply.

## References

[1] Nordic Council of Ministers. Nordic Nutrition Recommendations 2012: integrating nutrition and physical activity. Nordic Nutrition Recommendations. Report No: 5. Copenhagen: Nordic Council of Ministers; 2014. doi: 10.17226/11537

[2] Institute of Medicine. Dietary reference intakes: the essential guide to nutrient requirements. Washington, DC: The National Academies Press; 2006. doi: 10.17226/11537

[3] Penberthy WT, Kirkland JB. Niacin. In: Marriott BP, Birt DF, Stalling VA, Yates AA, eds. 11th ed. Present knowledge in nutrition: basic nutrition and metabolism. San Diego, CA: Elsevier Science & Technology; pp. 209–224.

[4] Covarrubias AJ, Perrone R, Grozio A, Verdin E. NAD^+^ metabolism and its roles in cellular processes during ageing. Nat Rev Mol Cell Biol 2021; 22(2): 119–41. doi: 10.1038/s41580-020-00313-x 33353981 PMC7963035

[5] Blomhoff R, Andersen R, Arnesen EK, Christensen JJ, Eneroth H, Erkkola M, et al. Nordic Nutrition Recommendations 2023. Copenhagen: Nordic Council of Ministers; 2023. doi: 10.6027/nord2023-003

[6] Christensen JJ, Arnesen EK, Andersen R, Eneroth H, Erkkola M, Høyer A, et al. The Nordic Nutrition Recommendations 2022 – principles and methodologies. Food Nutr Res 2020; Food Nutr Res 2020; 64: 4402. doi: 10.29219/fnr.v64.4402 PMC7307430 32612489

[7] Høyer A, Christensen JJ, Arnesen EK, Andersen R, Eneroth H, Erkkola M, et al. The Nordic Nutrition Recommendations 2022 – prioritisation of topics for *de novo* systematic reviews. Food Nutr Res 2021; 65: 7828 doi: 10.29219/fnr.v65.7828 PMC8897982 35291553

[8] Pallas Health Research and Consultancy, Eeuwijk J, Oordt A, Terzikhan N, Noordegraaf-Schouten MV. Literature search and review related to specific preparatory work in the establishment of Dietary Reference Values for Niacin, Biotin and Vitamin B6. EFSA Support Publ 2012; 9(12): 474. doi: 10.2903/sp.efsa

[9] Garg A, Sharma A, Krishnamoorthy P, Garg J, Virmani D, Sharma T, et al. Role of niacin in current clinical practice: a systematic review. Am J Med 2017; 130(2): 173–87. doi: 10.1016/j.amjmed.2016.07.038 27793642

[10] He YM, Feng L, Huo DM, Yang ZH, Liao YH. Benefits and harm of niacin and its analog for renal dialysis patients: a systematic review and meta-analysis. Int Urol Nephrol 2014; 46(2): 433–42. doi: 10.1007/s11255-013-0559-z 24114284

[11] Kulikova VA, Gromyko DV, Nikiforov AA. The regulatory role of NAD in human and animal cells. Biochemistry 2018; 83(7): 800–12. doi: 10.1134/S0006297918070040 30200865

[12] Minto C, Vecchio MG, Lamprecht M, Gregori D. Definition of a tolerable upper intake level of niacin: a systematic review and meta-analysis of the dose-dependent effects of nicotinamide and nicotinic acid supplementation. Nutr Rev 2017; 75(6): 471–90. doi: 10.1093/nutrit/nux011 28541582

[13] Nikas IP, Paschou SA, Ryu HS. The role of nicotinamide in cancer chemoprevention and therapy. Biomolecules 2020; 10(3): 477. doi: 10.3390/biom10030477 32245130 PMC7175378

[14] Okabe K, Yaku K, Tobe K, Nakagawa T. Implications of altered NAD metabolism in metabolic disorders. J Biomed Sci 2019; 26(1): 34. doi: 10.1186/s12929-019-0527-8 31078136 PMC6511662

[15] Rajman L, Chwalek K, Sinclair DA. Therapeutic potential of NAD-boosting molecules: the in vivo evidence. Cell Metab 2018; 27(3): 529–47. doi: 10.1016/j.cmet.2018.02.011 29514064 PMC6342515

[16] Sahebkar A. Effect of niacin on endothelial function: a systematic review and meta-analysis of randomized controlled trials. Vasc Med 2014; 19(1): 54–66. doi: 10.1177/1358863x13515766 24391126

[17] Schandelmaier S, Briel M, Saccilotto R, Olu KK, Arpagaus A, Hemkens LG, et al. Niacin for primary and secondary prevention of cardiovascular events. Cochrane Database Syst Rev 2017; 6(6): Cd009744. doi: 10.1002/14651858.CD009744.pub2 28616955 PMC6481694

[18] Wan P, Moat S, Anstey A. Pellagra: a review with emphasis on photosensitivity. Br J Dermatol 2011; 164(6): 1188–200. doi: 10.1111/j.1365-2133.2010.10163.x 21128910

[19] Combs GF, McClung JP. The vitamins: fundamental aspects in nutrition and health. 5th ed. Amsterdam: Elsevier/Academic Press, 2017.

[20] Kirkland JB. Niacin. In: Ross AC, Caballero B, Cousins RJ, Tucker KL, Ziegler TR, eds. Modern nutrition in health and disease. 11th ed. Philadelphia, PA: Wolters Kluwer Health/Lippincott Williams & Wilkins; 2014, pp. 331–340.

[21] Penberthy WT, Caudill MA. Niacin, riboflavin, and thiamin. In: Stipanuk MH, Caudill MA, eds. Biochemical, physiological, and molecular aspects of human nutrition. 4th ed. Saint Louis, MO: Elsevier; 2019, pp. 591–613.

[22] Cantó C, Menzies KJ, Auwerx J. NAD(+) metabolism and the control of energy homeostasis: a balancing act between mitochondria and the nucleus. Cell Metab 2015; 22(1): 31–53. doi: 10.1016/j.cmet.2015.05.023 26118927 PMC4487780

[23] Fang EF, Lautrup S, Hou Y, Demarest TG, Croteau DL, Mattson MP, et al. NAD^+^ in aging: molecular mechanisms and translational implications. Trends Mol Med 2017; 23(10): 899–916. doi: 10.1016/j.molmed.2017.08.001 28899755 PMC7494058

[24] Katsyuba E, Romani M, Hofer D, Auwerx J. NAD^+^ homeostasis in health and disease. Nat Metab 2020; 2(1): 9–31. doi: 10.1038/s42255-019-0161-5 32694684

[25] Zapata-Pérez R, Wanders RJA, van Karnebeek CDM, Houtkooper RH. NAD^+^ homeostasis in human health and disease. EMBO Mol Med 2021: e13943. doi: 10.15252/emmm.202113943 34041853 PMC8261484

[26] Dietary reference intakes for thiamin, riboflavin, niacin, vitamin B6, folate, vitamin B12, pantothenic acid, biotin, and choline. Washington, DC: Institute of Medicine (IoM), Food and Nutrition Board, National Academy Press; 1998. 23193625

[27] EFSA NDA Panel (EFSA Panel on Dietetic Products, Nutrition and Allergies). Scientific opinion on dietary reference values for niacin. EFSA J 2014; 12(7): 3759, 42 pp. doi: 10.2903/j.efsa.2014.3759 PMC13136993 42087961

[28] Scientific Committee on Food. Opinion of the Scientific Committee on Food on the Tolerable Upper Intake Levels of Nicotinic Acid and Nicotinamide (Niacin) (expressed on 17 April 2002). European Commission, Health and Consumer Protection Directorate General. Brussels: Scientific Committee of Food; 2002.

[29] EFSA Panel on Nutrition, Novel foods and Food allergens (NDA), Turck D, Castenmiller J, de Henauw S, Hirsch-Ernst KI, Kearney J, et al. Safety of nicotinamide riboside chloride as a novel food pursuant to Regulation (EU) 2015/2283 and bioavailability of nicotinamide from this source, in the context of Directive 2002/46/EC. EFSA J 2019; 17(8): e05775. doi: 10.2903/j.efsa.2019.5775 32626405 PMC7009190

[30] Bakaloudi DR, Halloran A, Rippin HL, Oikonomidou AC, Dardavesis TI, Williams J, et al. Intake and adequacy of the vegan diet. A systematic review of the evidence. Clin Nutr 2021; 40(5): 3503–21. doi: 10.1016/j.clnu.2020.11.035 33341313

[31] Neufingerl N, Eilander A. Nutrient intake and status in adults consuming plant-based diets compared to meat-eaters: a systematic review. Nutrients 2021; 14(1): 29. doi: 10.3390/nu14010029 35010904 PMC8746448

[32] Bieganowski P, Brenner C. Discoveries of nicotinamide riboside as a nutrient and conserved NRK genes establish a Preiss-Handler independent route to NAD^+^ in fungi and humans. Cell 2004; 117(4): 495–502. doi: 10.1016/s0092-8674(04)00416-7 15137942

[33] Lemming EW, Pitsi T. The Nordic Nutrition Recommendations 2022 – food consumption and nutrient intake in the adult population of the Nordic and Baltic countries. Food Nutr Res 2022: 66. doi: 10.29219/fnr.v66.8572 PMC9199833 35757440

[34] Powers HJ. Current knowledge concerning optimum nutritional status of riboflavin, niacin and pyridoxine. Proc Nutr Soc 1999; 58(2): 435–40. doi: 10.1017/S0029665199000579 10466188

[35] EFSA Scientific Committee, Hardy A, Benford D, Halldorsson T, Jeger MJ, Knutsen KH, et al. Update: guidance on the use of the benchmark dose approach in risk assessment. EFSA J 2017; 15(1): 4658, 41 pp. doi: 10.2903/j.efsa.2017.4658 PMC7009819 32625254

